# Porosity Measurements and Analysis for Metal Additive Manufacturing Process Control

**DOI:** 10.6028/jres.119.019

**Published:** 2014-09-16

**Authors:** John A Slotwinski, Edward J Garboczi, Keith M Hebenstreit

**Affiliations:** 1 National Institute of Standards and Technology, Gaithersburg, MD 20899; 2 Binghamton University, State University of New York Binghamton, NY 13902

**Keywords:** additive manufacturing, Archimedes, cobalt-chrome, Direct Metal Laser Sintering, porosity, powder bed fusion, ultrasonic NDT, X-ray computed tomography

## Abstract

Additive manufacturing techniques can produce complex, high-value metal parts, with potential applications as critical metal components such as those found in aerospace engines and as customized biomedical implants. Material porosity in these parts is undesirable for aerospace parts - since porosity could lead to premature failure - and desirable for some biomedical implants - since surface-breaking pores allows for better integration with biological tissue. Changes in a part’s porosity during an additive manufacturing build may also be an indication of an undesired change in the build process. Here, we present efforts to develop an ultrasonic sensor for monitoring changes in the porosity in metal parts during fabrication on a metal powder bed fusion system. The development of well-characterized reference samples, measurements of the porosity of these samples with multiple techniques, and correlation of ultrasonic measurements with the degree of porosity are presented. A proposed sensor design, measurement strategy, and future experimental plans on a metal powder bed fusion system are also presented.

## 1. Introduction

Unlike traditional manufacturing processes such as turning and milling that produce parts by removing unwanted material from a larger piece, additive manufacturing (AM) processes build parts one thin layer at a time. This can be done in a variety of ways, such as sintering of powder via laser or electron beams, extrusion and deposition of polymer via a heated orifice, or selective curing of liquid photopolymers. These processes can all produce complex, high-value parts that cannot be fabricated with traditional material removal processes, can accomplish this without tooling, and with the ability to go almost directly from a digital design to part. The full vision of additive manufacturing includes using these processes to produce complex, customized metal parts for use in high-stress, mission-critical aerospace applications, such as jet engine components and turbine blades, where innovative, weight-saving part designs that include complex interior structures could revolutionize the manufacturing industry.

Additive manufacturing successes have received significant attention in the popular press in the last couple of years, and while additive manufacturing is already producing customized metal parts in niche applications such as dental implants [1], and demonstrating truly impressive capabilities that generate lots of societal excitement for the future of AM [2,3], the full benefits of additive manufacturing are not yet realized in a widespread way across the manufacturing industry. This is due to several technical challenges that must first be overcome, including a lack of process understanding and a lack of *in-situ* process monitoring and control, especially in metal AM systems [4,5]. A few commercial AM systems do purport to have built-in *in-situ* process monitoring and control [6,7], but this is rare.

The amount of porosity within parts produced via AM is an area of interest in the AM community. Parts destined for high stress applications should be fully-dense, so as to minimize the possibility of part failure during service. On the other hand, a degree of porosity, especially surface-breaking porosity, is sometimes desirable and can be intentionally engineered into certain bio-medical implants, since the pores promote better osseointegration with biological tissue [8]. These requirements highlight the need for a real-time *in-situ* porosity monitoring capability, where the absolute level of porosity in a part can be measured and controlled while it is being built. In addition, even relative measurements of the changes in the porosity of a part during fabrication provides useful process information, since it may be an indication that the AM process is adversely changing and needs real-time adjustment.

In this paper we present foundational research aimed at developing a real-time, *in-situ* ultrasonic sensor for monitoring changes in porosity in metal cobalt-chrome (CoCr) parts during additive manufacturing, for process monitoring. Previous theoretical models showing the relationship between the ultrasonic wavespeed in a material and the degree of porosity in that material are presented, and are used to establish the measurement precision required to detect small changes in porosity. The construction and ultrasonic measurements of CoCr reference samples, made via an additive manufacturing powder bed fusion process, are described. Several different methods for measuring the samples’ density (and hence their porosity) are applied and compared, since accurate and precise measurements of wavespeed and porosity are necessary to be able to develop an accurate and precise relationship between the two. The strengths and weaknesses of these distinct techniques for measuring porosity are discussed. Finally, correlations between the part porosity and ultrasonic wavespeed measurements, along with a preliminary *in-situ* porosity sensor design for use in a metal powder bed fusion system, are presented.

## 2. Ultrasonic Technique for Wavespeed Measurements

Ultrasonic Non-Destructive Testing (NDT) techniques provide a non-damaging means of measuring the properties of solids, and have principal applications for defect detection, part thickness measurements, and determination of material properties such as elastic moduli [9] to a high precision and with low uncertainties [10]. The basic ultrasonic NDT techniques are rapid, have high penetration power and large sensitivity, and generally only require one-sided access to the specimen or material being measured [9].

There is a litany of previous theoretical and experimental work that explores the dependence of the ultrasonic wavespeed in a material on the amount of porosity within that same material. The theoretical work includes both linear dependencies - elastic theories that assume low levels of uniform spherical porosity - and non-linear dependencies - scattering theories that take into account the shape and orientation of pores [11–17]. Using several of these models, and assuming an approximate fully-dense longitudinal wavespeed of 6300 m/s for CoCr, we theoretically calculate that an absolute change in porosity of 0.2 % should be revealed by a change in longitudinal wavespeed of 20 m/s. This corresponds to an expected change in the measured time-of-flight for a 10 mm thick sample of 0.01 µs.

In this work, a variety of configurations were used to measure the ultrasonic wavespeed, including longitudinal wave pulse-echo time-of-flight (both contact and immersion), longitudinal through-transmission time-of-flight, and shear wave pulse-echo time-of-flight. All of these distinct configurations gave similar qualitative results. Only the longitudinal wave pulse-echo time-of-flight measurements with a contact transducer are presented here, since this sensor type and measurement method are the best candidate for use *in-situ* in a metal powder bed fusion system. Here a commercial 5 MHz contact transducer with a piezoelectric element with a nominal diameter of 12.7 mm is used. The ultrasonic signals were generated and received with a commercial 30 MHz bandwidth pulser-receiver that employed a shock-impulse excitation. The received signals (echoes) were fed into a digital oscilloscope that had a 300 MHz bandwidth and a sampling rate of 2.5 Giga Samples per second. Three independent measurements were performed on each CoCr porosity sample (described in Sec. 3). The transducer was coupled to the center of the top surfaces of each porosity sample by a thin layer of water-soluble gel couplant, and the transducer was re-seated on the sample between each measurement. The time record was averaged a minimum of 512 times, and the round trip time of flight through the sample was measured by using the oscilloscope’s cursors to measure the time between the first positive peaks of the first and second back-wall echoes of the averaged signal. This method is equivalent to the traditional pulse-echo-overlap technique reported elsewhere [10]. The round trip travel time for the ultrasonic signals was measured to a resolution of 0.005 µs, which is better than that required to detect an absolute change of 0.2 % porosity in a 10 mm thick sample, and is certainly theoretically precise enough to detect absolute changes of 0.5 % and larger. The results of these measurements are shown in Sec. 4.7.

## 3. Porosity Reference Samples

Empirically correlating ultrasonic wavespeed measurements with material porosity requires reference samples that have independently-characterized amounts of porosity. A total of sixteen CoCr disks with nominal diameters of 40 mm and thicknesses of 10 mm were produced by a commercial additive manufacturing service bureau, in three separate builds, using an EOS M270 Direct Metal Laser Sintering System (DMLS)^1^.

The approach chosen for this effort was to start with a developmental build, varying both the hatch speed and hatch spacing to determine the porosity response of the material to these laser scan parameters. In order to ensure consistency throughout the samples, a single hatch scanning style (e.g., skin exposure) was employed for the entire part. This first build produced six disk samples with scan parameters as shown in Table 1. The porosity levels of the samples from this first build were provided by the service bureau, as determined by simple mass and optical methods.

Based on the results, it was clear that increasing either hatch speed or hatch spacing would increase the porosity level. In order to have the greatest resolution, hatch spacing was chosen to be the control variable. Hatch speed can be varied by 1 mm/s with a nominal value of 800 mm/s, while hatch spacing could be varied only by 0.01 mm with a nominal value of 0.10 mm. Using the results for samples 1, 2, and 3 with constant hatch spacing, a simple quadratic model for porosity as a function of hatch speed was developed. This model was used for the two subsequent builds that were run with the goal of producing samples with porosity levels at from 0 to 5 % by varying the hatch speed.

Two of the sixteen disks had porosity levels that were so large that the back wall ultrasonic reflected signals were not detectable. Further measurements on these samples were not performed. The metal powder used to make these parts is commercially available from the machine vendor. A full treatment of the measured characteristics of this type of powder can be found elsewhere [18]. The powder had measured d(0.1), d(0.5), and d(0.9) values of 12.3 µm, 25.0 µm, and 45.0 µm, respectively, as determined using a commercial laser diffraction system [18]^2^.

The nominal physical properties and elemental abundance of parts made from this powder on an M270 system using the standard vendor-provided CoCr build parameters are shown in Table 2 [19].

Since subsequent porosity calculations required the bulk CoCr density, pycnometry measurements were performed on CoCr powder that was nominally identical to that used in the production of these disks. Helium pycnometry [20], using a commercial instrument, was used to measure the density of the metal powders, which is assumed to be the same density of a fully dense built part that has no discernible porosity. These measurements were performed as follows: an empty container was used to tare a mass balance. The metal powder was added to fill the cell, lightly tamped, and the mass of the powder determined. In the helium pycnometer, the amount of helium that fills the empty volume around the powder is determined by using the measured temperature and pressure of the helium in the cell and the ideal gas law, which is very accurate for helium at room temperature and pressure. Since the empty cell volume is precisely known, by using the pycnometer on the empty cell, the difference between the two volumes is the actual volume of the powder. A simple quotient gives the powder density, averaged over all the particles present. If some of the particles are porous, but the pores are accessible from the surface, then the true metal density is still determined. If there are hollow particles such that some pores in the particles are not accessible from the surface by the helium atoms, then these pores will be considered part of the powder and thus the determined powder density will be somewhat smaller than the actual metal density. The X-ray Computed Tomography (X-ray CT) scans reported in Sec. 4.4 also showed very little evidence for hollow particles.

The measurement run for this powder took approximately 30 min. Judging by past experiences with other kinds of powders that did have internal porosity, as verified by Scanning Electron Microscopy (SEM) and X-ray CT, if the metal particles had significant internal porosity the run time would have been longer for the helium to penetrate the particles. Using this technique gave a CoCr density of 8.3046 g/cm^3^ ± 0.0013 g/cm^3^ where the measurement uncertainty has a k = 2 coverage factor [21].

## 4. Measurements and Results

**Fig. 1:**
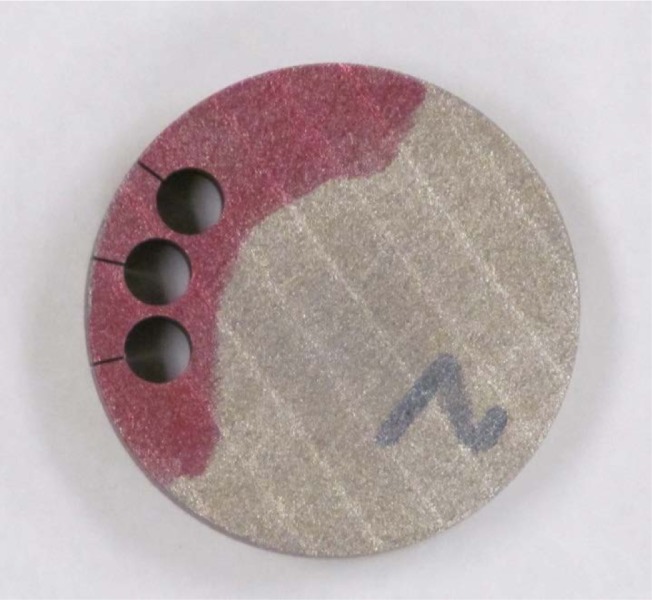
Sample 3–2, showing where the 5 mm cylinders were cut out via electric discharge machining.

The unaltered disk samples were used for both bulk porosity measurements (Sec. 4.1) and ultrasonic wavespeed measurements (Sec. 4.7). Other measurement methods reported here such as the Archimedes, X-ray CT, and local porosity measurements used smaller samples. These were made out of the large disks by using Electric Discharge Machining to remove small, 5 mm diameter cylinders from the disks. A minimum of three cylinders were cut from each disk, roughly 2 mm in from the sample edge, and had the same height as the disk from which they were being cut (roughly 10 mm). This is shown in Fig. 1. The distance from the center of a given hole to the outer surface of the full sample, going along a diameter of the full sample, is about 5 mm, so that the solid material neck thickness between the outer surface and the edge of one of the cylinders is about 2.5 mm.

For identification purposes in this paper the various samples use the notation *set-disk cylinder*, where *set* is the build identifier (1–3), *disk* is the disk identifier (1–5), and *cylinder* identifies the cylindrical sub-specimens cut from the disks (A–E). For example, the notation 2–4B refers to the ‘B’ cylinder cut out of disk #4 from the second build.

Following all of the measurements reported in this paper, the disks were cut into two pieces, using a water-fed abrasive saw with a water-soluble machining coolant. The disks’ outer and interior cross-sectional surfaces were then examined using digital imaging techniques to confirm the presence of pores and to develop quantitative digital imaging measurements of porosity. These results will be presented at a later date.

### 4.1 Bulk Mass Measurements of Porosity

In this method, a measurement of the specimen density, and hence its porosity since the full-dense density is known (Sec. 3), is accomplished through basic mass and volume measurements and the density-mass-volume relationship. This measurement is simple to implement, and uses commonly available measuring equipment, but results in a measured porosity that is averaged throughout the entire part, and is not sensitive to local variations in part porosity. The main uncertainty is in the volume measurements, since we assume that the full samples are perfect cylinders. This uncertainty far outweighs the uncertainty in measuring the mass.

Before the cylinders were removed from the disks, the disks’ masses were measured using a digital scale, and the disks’ diameters and thicknesses were measured using a micrometer. Three measurements were performed for each measured quantity. From these dimensional measurements, the disk volume was computed. The measured mass and volume of each disk was then used to compute the disk density, and from this, using the measured CoCr density of 8.30 g/cm^3^ reported above in Sec. 3, the porosity was calculated. These results are summarized in Table 3. A discussion and summary of the methods and calculations used to determine the measurement uncertainties reported in this paper are contained in Appendix A.

### 4.2 Localized Mass Measurements of Porosity

This method is identical to that of Sec. 4.1, except that the mass and dimensional measurements were performed on the small cylinders cut out of each disk. This allows for localized measurements of porosity. These results are shown in Table 4. Note that the additional significant figures, compared to Sec. 4.1 and Table 3, are the result of using a mass scale with a higher precision that is designed for lighter weight samples. Note the scatter between A, B, and C 5 mm cylinders. This could indicate that the pore structure in some cases varied on this length scale.

### 4.3 Archimedes Measurements of Porosity

The Archimedes method for determining the density (and hence the porosity) of a material is a classic method that has been used previously for determining the density of materials made via additive manufacturing [22]. While it is relatively simple to perform with commercial instrumentation, samples with surface breaking pores or cracks that allow for water-infiltration may result in erroneous measurements.

For the Archimedes measurements, the measured cylinder density is given by

$$\rho =\left(\frac{M_{a}}{M_{a}-M_{w}}\right)\rho_{W}$$ (1)

where *M_a_* is the measured mass of the cylinder (average of three independent measurements) measured in air, *M_w_* is the measured mass of the cylinder (average of three independent measurements) measured in water, and *ρ_W_* is the density of water, which we assume to be 1.0 g/cm^3^. While in reality the density of water changes with temperature, it varies by only 2 parts per thousand over the range 15 °C to 25 °C [23]. As such we treat this variation as negligible, especially relative to the other uncertainties in our measurements.

For these measurements, we used a commercially available Mettler AT201 measurement system with distilled water, to minimize the presence of air bubbles in the water, at room temperature (roughly 22 °C). The water was in thermal equilibrium with the laboratory air. This system allows for precise mass measurements of small samples in both water and air. Three measurement trials were performed for each cylinder both in air and in water. Some care was taken to ensure that air bubbles were not present on the samples under test, although smaller ones could have been missed. The scale was re-zeroed before each measurement, and the enclosure was shut around the scale to prevent air currents from affecting the measurement. Each measurement result was recorded after the scale had reached equilibrium. The results of these measurements are shown in Table 5.

### 4.4 X-Ray Computed Tomography (X-Ray CT) Measurements of Porosity

X-ray computed microtomography is a technique that obtains X-ray images through a sample as it is rotated. The specimen is subjected to X-rays from many angles by rotating the specimen through many (of order 1000) small angular increments between 0 and 360° [24]. Reconstruction algorithms yield a sequence of 2-D gray level images (slices) perpendicular to the vertical axis of the cylindrical specimens that represent differences in the attenuation (which is dependent mostly on density) of different points within it. These slices can be computationally stacked to yield a 3-D view of the specimen. This 3-D image stack has a typical size of 1000 × 1000 × 1000 voxels. If each voxel is a 2-byte integer, this gives a 2 Gbyte data size for the image stack. X-ray CT is a completely non-destructive method for seeing inside a sample, at voxel sizes down to tens of nanometers. As long as the X-rays can completely penetrate the sample with a minimum of about 10 % transmission, and there is some difference in absorption between different parts of the sample, good images can be formed. If voxel sizes of the order of micrometers are desired, this limits the size of the sample that can be used. The full CoCr cylindrical samples could not be imaged, which is why 5 mm diameter cylinders were cut from the full specimens.

The X-ray CT instrument that produced the results presented in this paper was an Xradia Versa, which has a maximum voltage 160 kV and maximum power 10 W. In all cases, an inner portion of the 5 mm cylinders was scanned, which produced a cylindrical image. The X-rays were able to penetrate the 5 mm cylinders and produced sharp images. All these images were 2-byte tiff files, with gray scale running from 0 to 65535.

X-ray CT has been used previously on samples built using additive manufacturing [25]. In that work, a voxel size of 9 µm was used, which is significantly larger than the voxel sizes used in this paper, to quantify porosity.

#### 4.4.1 X-Ray CT – Total Porosity Analysis

X-ray CT scans were made on at least one 5 mm cylindrical sample from each of the 14 disks. One 5 mm cylinder was chosen from each test sample to measure the pore structure in a Xradia Versa X-ray CT unit. Settings of 155 kV for the X-ray source voltage, operating at 10 W of power, was used for each sample. The thickest filter available on the instrument was employed so as to screen out the lower energy photons that could not penetrate the sample. Each scan took approximately 5 h, taking an image at about 3000 angles in the X-ray CT process. Disks 1–2 and 1–5 had similar scans on two different 5 mm cylinders of the set, in order to give some idea of porosity variation on this length scale. The calculated standard deviation in Table 6 was over layers in the z direction, which is the build direction in the M270 DMLS additive system used to make these disks.

Each sample was mounted using polymeric glue on a steel or aluminum nail head and the nail was then placed in a pin vise sample holder. The scan was carried out, the slices reconstructed, and 16 bit tiff files were generated for further image analysis. There were about 1000 slices generated for each scan, but the top and bottom 50 slices were discarded because of X-ray cone-beam imaging artifacts [26]. Image analysis was applied to the remaining 900 or so images by using a simple gray scale threshold (white = solid, black = pore), which was chosen to be large enough such that the thresholded images had a qualitative visual match to pore structure, but low enough so as to minimize the amount of random black pixels generated in what was clearly the solid part of the image. This is an independent measure of porosity, but it is not as accurate as the other methods discussed in this paper, since there was a degree of subjectivity in choosing the threshold.

Since these scans were taken inside the 5 mm cylinders, the image was perfectly cylindrical. The background outside of the cylindrical microstructure image was given a third gray scale and so was not counted in the porosity analysis. The images were all about 1000 × 1000 pixels in size. The data in Table 6 were generated by averaging over the z direction only, which was normal to the slices and along the axis of the cylinder. The variation along this axis gave the standard deviation of the porosity. If stacked together, the images are approximately 2.4 mm in diameter and 2.1 mm in height. Each 5 mm cylinder represents about 1.6 % of the total sample volume for each disk. The actual volume of each individual scan, however, is equal to about 0.1 % of the total sample volume.

Comparing the standard deviation of the porosity of each layer, divided by the average porosity of the entire sample, gives an idea of how much the porosity fluctuated during the build, which may perhaps give an indication of how well-controlled the manufacturing process was during a particular build at the particular settings used. Using an X-ray CT examination of the porosity, in a post-process analysis, might be used as a quality control standard procedure for the control of the manufacturing process.

Two of the 5 mm samples, 1–1C and 3–1A, were found by the X-ray CT to have no measurable pores that could be seen using a 2.4 µm pixel size. There could have been pores several times smaller than this that did not show up in the X-ray CT images. Judging by this, they were deemed to be fully dense, which was the target porosity for these two samples.

**Fig. 2:**
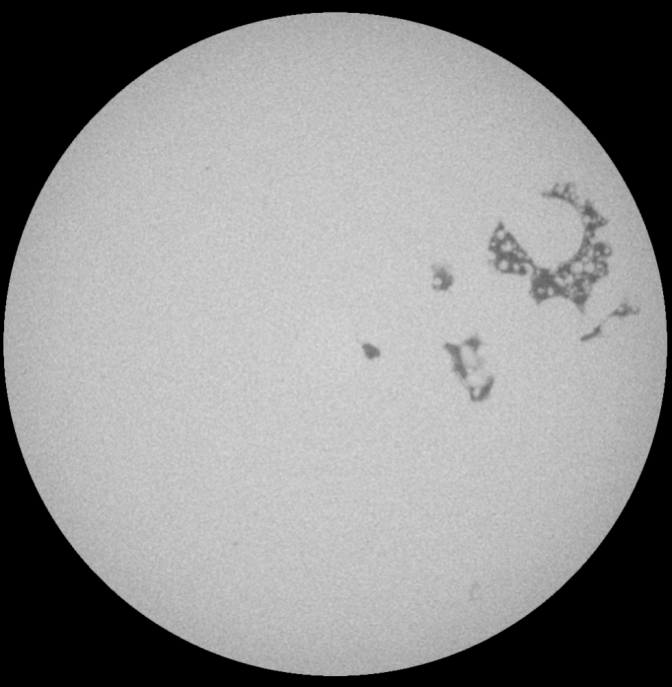
One slice from sample 2–1C 5 mm diameter cylinder, showing a very large defect. The full width of the image is about 2.4 mm.

The target porosity for sample 2-1, like the Sample 1 specimens for the other two sets, was 0.0 or full density. Taking a closer look at sample 2-1, we see a non-zero average density of 0.57 %, and the standard deviation is 0.348 %. The ratio of standard deviation to average gives a measure of how widely the porosity fluctuates from slice to slice, and for this case the ratio is equal to 0.61, which is the highest for all the5 mm cylindrical samples. Figure 2 shows a slice about half way through the set of slices studied, showing a very large pore compared to most pores observed. The porosity of this slice was about 2.3 %, which was the largest for this collection of slices. So this specimen is mostly fully dense, but with one or more large defects. This has negative implications for the strength, which would be higher if the porosity was more evenly distributed. In general, the relatively large magnitude of the standard deviations with respect to the average porosities agree with the results found in Ref. [25].

#### 4.4.2 X-Ray CT - More Detailed Analysis

X-ray CT scans were taken at two different voxel sizes of samples 1–2D and 1–5D. These two samples typified the low porosity and high porosity type of samples seen in Table 6. The two voxel sizes were about 0.8 µm/voxel (High resolution) and 2.5 µm/voxel (Low resolution). More exact numbers are given in Table 7. The scans were taken of volumes inside each sample. Each stack of 800–850 images was approximately 1000 × 1000 pixels, so that the physical size of the X-ray CT scans was approximately 0.68 mm in height and 0.8 mm in diameter, and 2.1 mm in height and 2.5 mm in diameter, for high resolution and low resolution, respectively.

Having the porosity per slice means that much more analysis can be performed: porosity per layer and how this varies, in all three principal directions; the standard deviation of porosity in any one of the three coordinate axis dimensions; the pore size distribution for sample 1–2, which did not have a connected pore space; the degree of connectivity for a connected porespace, like in sample 1–5; and a measure of anisotropic pore shape for isolated pores.

We will first discuss the general features of each sample as obtained from the image sets detailed in Table 7.

In Table 7, note that the average porosity was somewhat different between the low and high resolution scans for each sample, but the difference was within the standard deviation. The difference between the two values was not due to the higher resolution scan picking up pores that were not seen on the lower resolution scans, since the measured porosity was actually less, but was due to the spatial dependence of the microstructure. The higher resolution scans images less of the microstructure, so was more likely to have larger fluctuations [27].

**Fig. 3:**
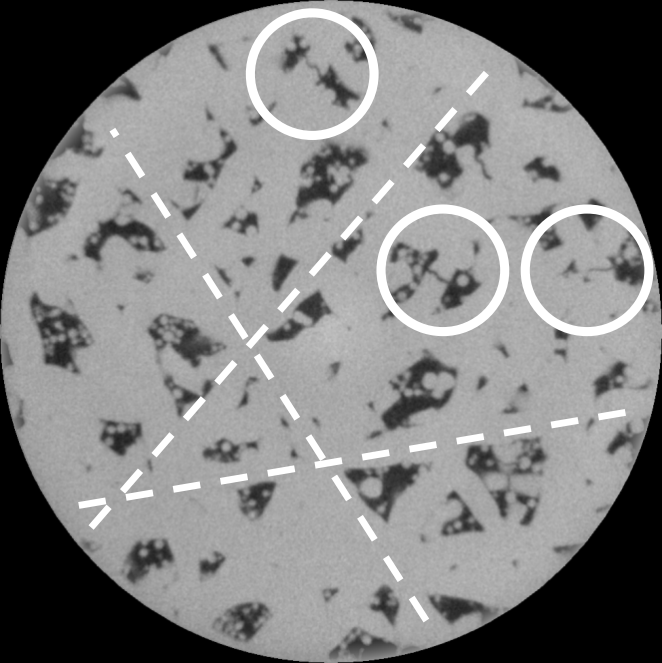
Sample 1–5D, X-ray CT of the interior of a 5 mm diameter cylinder cut from the original sample. The 5 mm cylinder had the same height as the original sample, but the image stack only extends part way through the sample. The pixel size in this image is 2.44 µm. Table 7 gives more details about the images taken from this sample. The white circles mark apparent cracks and the dashed white lines indicate laser tracks.

Figure 3 shows one slice of the sample 1–5D scan, imaged at 2.44 µm per pixel, so that the image size is about 2.4 mm. The gray scales in this image are those produced by the scanning process and reconstruction algorithm. One can clearly see the tracks of the laser as it traversed the system at approximately 0°, 60°, and 120° – the dashed white lines show three of these lines. Therefore, the larger pores seen are about 0.2 mm in size in this view, and there are many pores close to this size. Note that the pores are filled with metal powder, which was not sintered and therefore not or only tenuously merged into the main solid body in the manufacturing process. The white circles mark three different cracks, which appear to be actual cracks across solid material that was formed in the laser tracks, and not just indicating an incomplete joining of two or more powder particle aggregates.

**Fig. 4:**
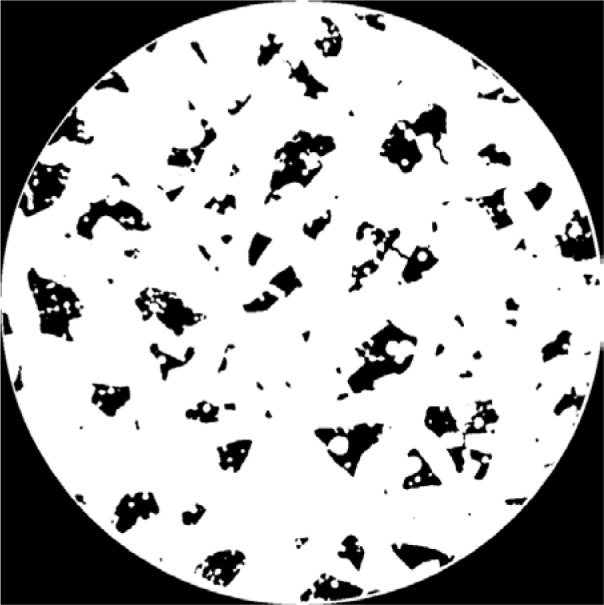
Same as Fig. 3 but thresholded into solids (white) and pores (black).

For comparison, Fig. 4 shows the same image but now thresholded at a gray scale of 32 000, which means all pixels with gray scales above 32 000 became white, and all others black. Careful comparison of Figs. 3 and 4 will show a close match in feature size. Note the apparent thin white “shell” that can be seen on the edge of the cylinder next to a large pore. This layer comes from the rest of the sample, which lies outside this cylindrical “virtual core” through the complete specimen. Also note that the rightmost crack, marked with a circle in Fig. 3, does not show up in Fig. 4 – this is an artifact of the thresholding, which affects the determined porosity.

**Fig. 5:**
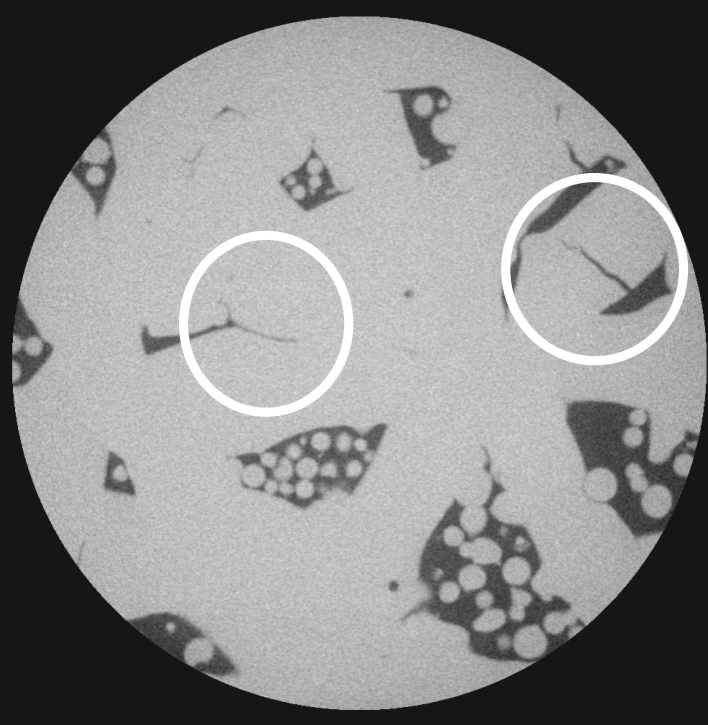
Sample 1–5D, X-ray CT of the interior of a 5 mm diameter cylinder cut from the original sample. The 5 mm cylinder had the same height as the original sample, but the image stack only extends part way through the sample. The pixel size in this image is 0.87 µm. Table 7 gives more details about the images taken from this sample. The white circles mark apparent cracks.

Figure 5 is a typical slice of sample 1–5D but with a smaller pixel size of 0.87 µm. The right-hand circle marks what is clearly a crack in the solid metal in a laser track, while the left-hand circle marks a site that could be a line where two sintering fronts have met and stopped at a small angle, so that there is a crack-like separation between the two fronts. Note that the apparent crack marked by the circle to the right narrows towards its end and has some slight branching structure at its tip, while the other feature is more uniform throughout its length. This difference in the two features suggests the above interpretation. The spherical particles remaining inside the pores vary in size but are consistent with the original particle size distribution of the cobalt chrome powder [18]. Careful inspection of Fig. 5 will show other such examples of these two kinds of features.

**Fig. 6:**
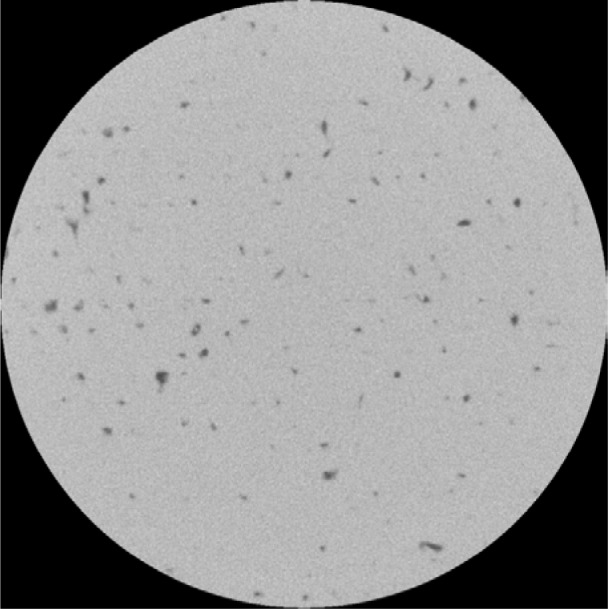
Sample 1–2D, X-ray CT of the interior of a 5 mm diameter cylinder cut from the original sample. The 5 mm cylinder had the same height as the original sample, but the image stack only extends part way through the sample. The pixel size in this image is 2.54 µm. Table 7 gives more details about the images taken from this sample.

Figure 6 shows a slice from sample 1–2D imaged at the lower resolution (larger pixel size). The porosity is much smaller than for sample 1–5D (see Table 7), and the average pore size is clearly also much smaller, too, than for sample 1–5D. The actual number of visible pores, comparing Fig. 6 and Fig. 3, does not seem to be that much different, however.

**Fig. 7:**
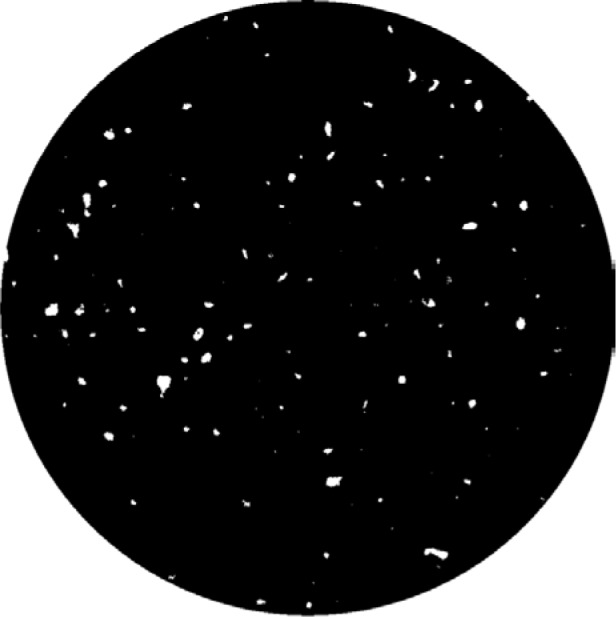
Same as Fig. 6 but thresholded into solid (black) and pores (white).

Figure 7 shows the same slice as in Fig. 6 but now thresholded into two phases, pores and solids. The black material outside the cylinder in Fig. 6 appears black in Fig. 7. The image was inverted compared to Fig. 4 so as to see the much smaller pores more clearly.

**Fig. 8:**
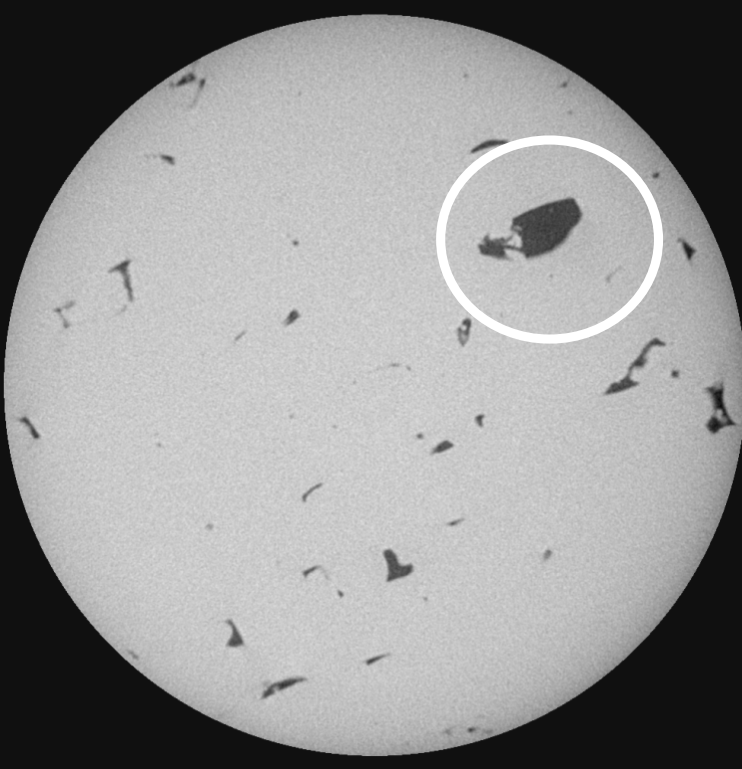
Sample 1–2D, high resolution.

Figure 8 shows a slice from the high resolution measurement of sample 1–2D. Compared to sample 1–5D results, one does not see many particles inside pores. Some are visible, but are not spherical and quite irregular, indicating a greater degree of sintering. The large pore that is marked by a circle has a longest dimension of about 0.1 mm. Most of the other visible pores are much smaller in volume than this pore, and usually smaller in all dimensions as well, although there do appear to be some crack-like pores, which are probably where two sintering fronts have collided to form a narrow pore. There is no visual evidence of cracks in this image, although an exhaustive search was not made of the other 850 slices in this image stack.

#### 4.4.3 Analysis of Porosity Variation in Structure

Since the X-ray CT image set shows the entire pore structure of this piece of the sample, it is possible to analyze how the porosity changes throughout the structure. A rectangular box-shaped piece was extracted from the center of the image sets listed in Table 7, approximately 620 × 620 × 851 (or 801) voxels. This is the largest such piece that still fits entirely within the cylindrical images. Then, starting in each direction, the porosity was computed for each slice and averaged, with the standard deviation computed over the slices. The average porosity in each direction, x, y, and z, must be the same, since the porosity over the same piece of microstructure is being totaled, but the standard deviation can be different depending on the direction. What kind of information could come from this analysis? Imagine a layered structure where each layer in the z direction alternates between two values of porosity. This would produce a non-zero standard deviation in the z direction but the x and y directions would have the same value porosity for each slice, the average of the two z-layer values. Therefore, in such a case, the conclusion is that the sample’s pore space was much more variable in the build direction, z, than in the other two, x and y.

Table 8 shows the variability of the porosity values obtained. For both samples, the standard deviation in the z direction is significantly larger than in the x and y direction, for the lower resolution scans. For the higher resolution scans, both y and z standard deviations are higher than the x value and are roughly equal. Since at lower resolution, one interrogates more of the actual microstructure, the numbers for the low resolution scans are to be taken more seriously than for the higher resolution scans. They show that there is more variability in the z direction, the direction in which layers are built up by the laser, than in the x and y directions within the plane of one laser-sintered layer.

**Fig. 9:**
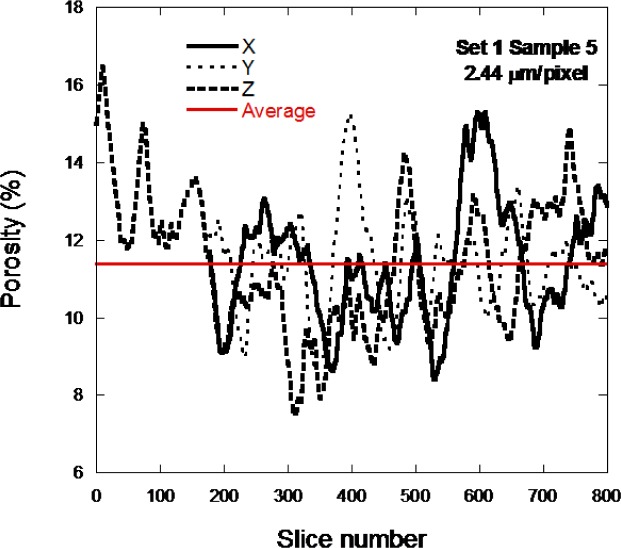
The porosity per slice is displayed for the sample 1–5D low resolution scan.

Figure 9 shows the porosity calculated in all three directions as a function of slice number for sample 1–5D, at low resolution, in order to see more of the actual microstructure. The x and y directions only have about 620 slices while the z direction has the full 851 or 801 slices through the thickness of the sample. The horizontal line shows the average porosity calculated for this piece of the pore structure. The porosity varies greatly throughout the sample. The z-curve does seem to deviate more widely from the horizontal line than do the x and y curves.

**Fig. 10:**
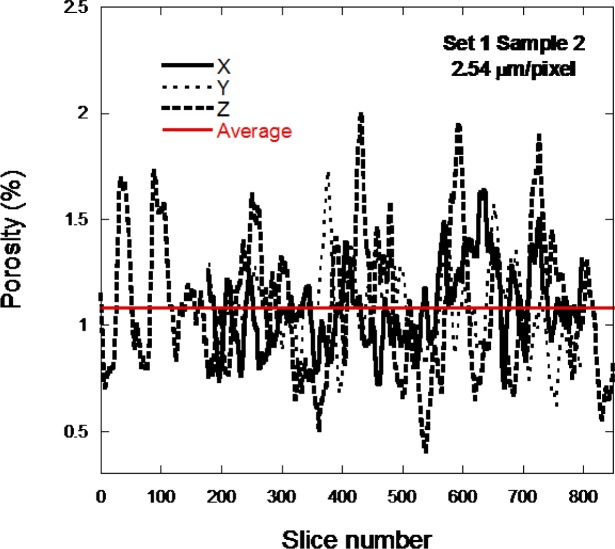
The porosity per slice is displayed for the sample 1–2D low resolution scan.

Figure 10 shows the porosity calculated in all three directions as a function of slice number for sample 1–2D, low resolution, in order to see more of the actual microstructure. The x and y directions only have about 620 slices while the z direction has the full 851 slices through the thickness of the sample. The horizontal line shows the average porosity calculated for this piece of the pore structure. The porosity greatly varies throughout the sample. The z-curve does seem to deviate more widely from the horizontal line than do the x and y curves.

It might be expected, since each layer is nominally identical in terms of what the laser is doing, that the porosity would be much more uniform, especially in the z-direction. However, it is important to remember that the average unsintered CoCr particle size was about 30 µm and the images are looking at a vertical scale that is much smaller than this. Therefore, variations in powder packing are probably causing these variations in porosity through the vertical layers of the samples. The variation shown in Figs. 9 and 10 mixes together the variations that come from laser control and the powder packing volumetric variations.

This analysis was repeated for sample 1–5D by averaging the porosity in the z-direction, using a 2.5 µm pixel size, over 10 and 20 layers (25 µm and 50 µm, respectively) and computing the standard deviation over these thicker layers. The computed standard deviation was essentially the same as that obtained by averaging over each layer. This may imply that the observed scatter in the data is not due to the powder packing randomness, but perhaps something fundamental in the process itself.

#### 4.4.4 Further Analysis of Isolated and Connected Pore Structures: Pore Shape and Size

Upon examining the images for sample 1–5D, it is clear that the porosity seems to be mainly isolated in the horizontal direction, with pores separated by solid metal in the laser tracks. However, for the same reason, the pores could very well be connected in the vertical direction. A burning algorithm [28] was applied to the complete set of images and it was found that the pore space was about 93 % percolated, which means that a connected cluster from top to bottom existed and 93 % of the pore volume was part of this connected cluster. This was true for both the high and low resolution image sets.

This means that practically all the pores form one pore, so that it is impossible to analyze individual pore size and shape. In the sample 1–2D images, however, the pores are clearly isolated in the x and y directions and turn out to be also isolated in the z direction, so that actual individual pores can be defined. Pores were identified down to 27 voxels in volume. Below this value, it was possible that several-voxel noise in the images would be identified erroneously as pores, so this cutoff was chosen. At the high resolution, there were about 1400 pores, while at the lower resolution, there were about 8100 pores identified.

To get a measure of pore shape and size, for each pore the largest length in the x, y, and z directions was computed and these lengths were averaged over the pores, weighted by pore volume. If the pores were close to being equi-axed, whether looking like cubes or spheres, these average lengths would be nearly equal. The standard deviation was computed for the average x, y, and z lengths, to give an idea of the width of the distribution. The actual distributions were quite noisy, since the number of pores was not large, and will not be shown.

Table 9 shows that the average pore length in the z direction, which is normal to the scans and in the same direction as the ultrasonic measurements were made, is significantly smaller than the lengths in the x and y directions, which are in the plane of the images. This implies that the pore shapes tend to be a bit flattened in the z direction, so the ultrasonic waves are seeing slightly oblate pores. Notice that this effect is more pronounced for the higher (smaller pixel size) resolution set of images. These images certainly resolve the pore shape more precisely than does the lower resolution images; however, there are more pores and thus better statistics for the low-resolution scans.

### 4.5 Elastic Simulations to Assess Mechanical Anisotropy

Anisotropy in mechanical strength would be a combination of anisotropy in elastic moduli and in flaw size and orientation. In this section, large-scale finite element computations are performed to try to get an idea of the degree of elastic anisotropy in these CoCr samples. A 400^3^ voxel piece of each image set was clipped out of its center and thresholded with the same values as were given in Table 7. Elastic moduli values of E = 200 GPa and *v* = 0.25 were assigned to the Cobalt Chrome voxels, which is approximately the Young’s modulus value found for pure CoCr alloy, while the value of Poisson’s ratio is a bit lower than the typical 0.3 value for many elemental metals. A finite element scheme, where each voxel is a tri-linear finite element, was applied to this structure. The program runs in parallel, using 200 processors [29–31]. Tables 10–13 show the composite moduli that were obtained for all four image sets. The caption of each table gives the values of K, G, E, and *v* that would be obtained if the elastic moduli tensor were spherically averaged.

The elastic moduli tensor elements that would appear in the blank sections of Table 10–13 were actually non-zero, but very small compared to the other tensor elements, so have been neglected. Table 10, for sample 1–5D, high resolution, clearly shows that while C_11_ ≈ C_22_, C_33_ is about 10 % smaller, similar to what was measured in stainless steel AM samples manufactured using a similar process, indicating approximate isotropy in the horizontal, x and y, direction, and somewhat smaller stiffness in the vertical, z direction. Examining Fig. 3, one sees an approximate hexagonal symmetry imposed upon the pore structure due the systematic variation of the laser tracks. It is known that hexagonal symmetry gives elastic isotropy [32]. However, for the other three systems listed in Tables 11–13, the elastic moduli tensor is approximately isotopic. So elastic anisotropy probably is not enough, by itself, to explain any strength anisotropy.

The elastic computations can also be used to get an idea of pore shape anisotropy. Consider the sample 1–2D image sets, for isolated pores with a total porosity of about 1 %. If the pores were all spheres, then one could use the exact result for the effect of spherical cavities, in the dilute limit of a few volume percent or less, on the overall elastic properties [31,33]. A convenient measure is the intrinsic elastic moduli [29,30], defined by K = K_o_ + K_o_[K]c_i_, where K is the composite bulk modulus, K_o_ is the matrix bulk modulus, c_i_ is the volume fraction of the inclusions, and [K] is the intrinsic bulk modulus. The intrinsic shear modulus, [G], is similarly defined. The intrinsic bulk and shear moduli give a measure of the effect of an inclusion, in this case a pore, on the composite elastic moduli. For a spherical pore, with the given cobalt chrome elastic moduli, [K] = −2.25 and [G] = −1.963. For sample 1–2D, at high resolution [K] = −3.31 and [G] = −2.55. For low resolution, [K] = −2.92 and [G] = −2.35. So the pores are definitely not spherical. If one assumes that they are ellipsoids, then using the exact formulas for ellipsoids [34,35], one has to go to an oblate ellipsoid shape with semi-axis ratios, with respect to the smallest semi-axis, of 1 - 3.5 - 3.5 to get this much change in the intrinsic elastic moduli away from the sphere result. The shape data in Table 8 does not suggest this kind of geometrical anisotropy in the pores, so the increase in magnitude of the intrinsic elastic moduli as compared with spherical pores is almost certainly due to the effect of corners and cracks, as was seen in Figs. 2–6.

The determination of these elastic constants is useful in the analysis of ultrasonic wavespeed as a function of porosity as shown in Sec. 4.7 since these elastic constants and the wavespeed are related. For an elastically isotropic material, C_11_ = K + 4/3 G, and for a dilute volume fraction of pores φ, added in random positions and orientation,

$$C_{11}={C_{11}}^{\circ }+{C_{11}}^{\circ }[C_{11}]\phi$$ (2)

where [C_11_] = [K] + 4/3 [G] and the superscript or subscript “o” stands for the matrix material.

For spherical pores, filled with zero moduli material [27,28],

[K]=−3(Ko+43Go)Go (3)

$$[G]=-\frac{5}{3}\frac{\left(K_{o}+\frac{4}{3}G_{o}\right)}{\left(K_{o}+\frac{8}{9}G_{o}\right)}$$ (4)

Now, the longitudinal ultrasound velocity is

$$v^{2}=\frac{c_{11}}{\rho }=\frac{c_{11}^{o}+c_{11}^{o}[c_{11}]\varphi }{\rho_{0}-\varphi \rho_{o}}$$ (5)

Expanding this for small values of ϕ and keeping only linear order terms in φ, we get

$$v^{2}={\frac{1}{\rho }}_{0}(C_{11}^{o}+C_{11}^{o}[C_{11}]\varphi )(1+\varphi )=\frac{c_{11}^{o}}{\rho_{0}}(1+[C_{11}]\varphi +\varphi )$$ (6)

and finally, taking the square root of both sides and again expanding for small values of ϕ,

$$v=v_{o}+\frac{v_{o}}{2}(1+[C_{11}])\varphi$$ (7)

For our system, if we assume that *v*_o_ is about 6250 m/s, then taking the manufacturer’s value of E = 220 GPa for the fully dense cobalt chrome alloy, and taking the Poisson’s ratio *v* = 0.33, similar to many elemental metals, gives a value of C_11_ which, along with the density of 8300 kg/m^3^, gives this fully dense longitudinal wave speed. In the equation above, ½ v_o_(1 + [C_11_]) is −130.67 m/s/%, which agrees well with the fitted slopes computed in Sec. 4.7.

The X-ray CT images and the shape analysis show that the pores are in general not spherical. However, at low porosities, the pores are close to being spherical due to the surface energetics of melting and resolidification. So at low porosities, 1 % to 2 %, the spherical pore result is probably theoretically correct. If the pores stayed spherical as porosity increased, the quadratic term in porosity, which is positive [36,37], would gradually exert itself and the slope would appear to become shallower (less negative), if still measured from the zero porosity limit. However, as the porosity increases the pores, as we have seen in the X-ray CT images, also become less spherical. The spherical pore gives the minimum slope for any shape pore [36,37], so that varying the pore shape will cause the magnitude of the negative slope to increase, making the velocity decrease faster with porosity. However, the magnitude of the positive quadratic term in porosity will also increase with porosity as the pores become less spherical. These two effects may counteract each other to some extent and may thereby cause the spherical pore linear slope to appear to fit the experimental results to higher porosities than would be warranted if the pores were only spherical at all porosities.

### 4.6 Comparison of All Porosity Results

**Fig. 11:**
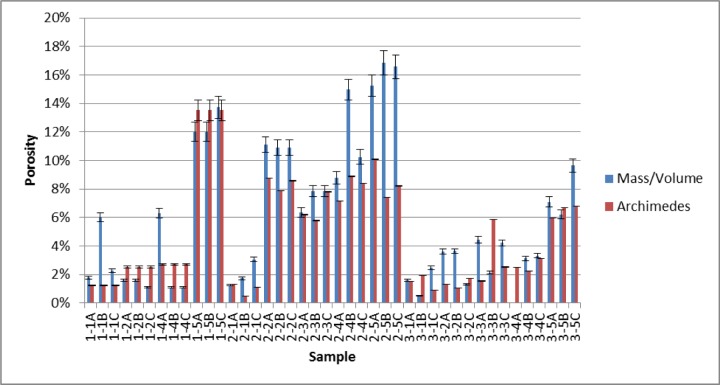
Measured porosity and 2σ measurement uncertainty bars for the cut out cylinders, as determined by both localized mass and volume measurements (Sec. 4.2) and the Archimedes method (Sec. 4.3).

**Fig. 12:**
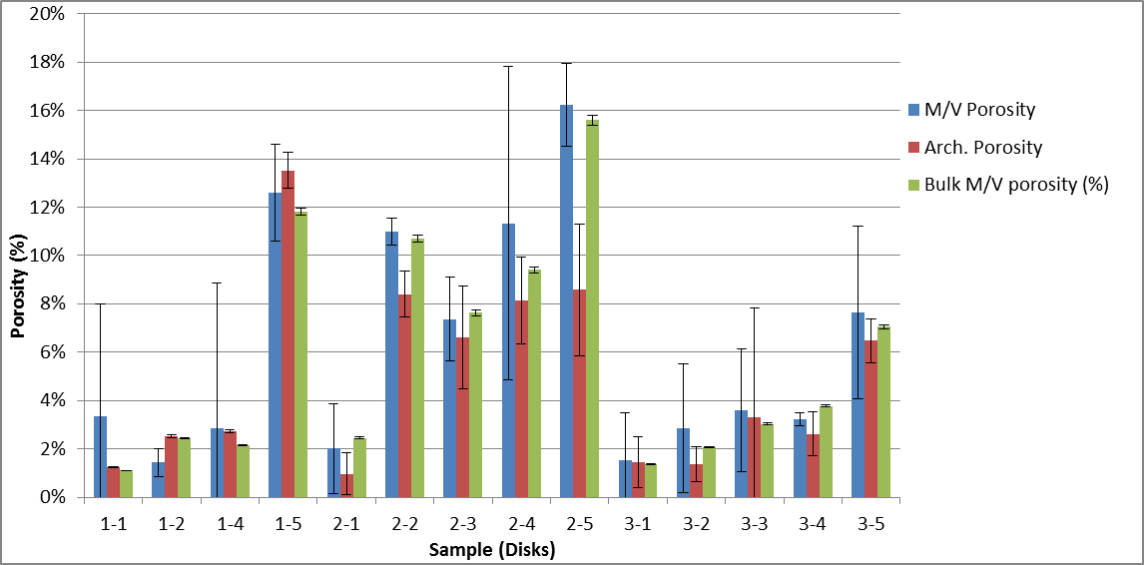
Overall disk porosity and 2σ measurement uncertainty bars determined from individual cylinder porosities (both mass/volume and Archimedes) and the bulk mass and volume of the disks (Sec 4.1).

**Fig. 13:**
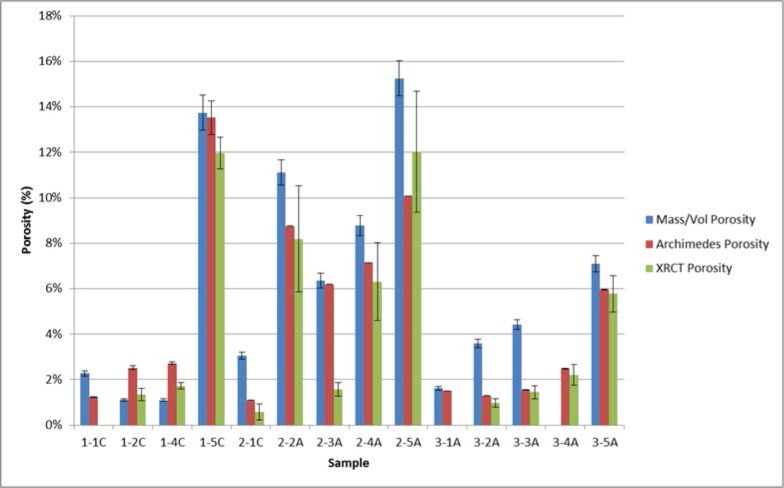
Comparison of the individual cylinder porosity results for those cases where all three methods (mass/volume, Archimedes, XRCT) were applied.

This section summarizes and compares the measured porosity values for both the bulk CoCr disks as well as the smaller cylinders that were cut out of the disks. Figure 11 shows the measured porosity and 2σ measurement uncertainty bars for the cut out cylinders, as determined by both localized mass and volume measurements (Sec. 4.2) and the Archimedes method (Sec. 4.3). The localized cylindrical porosity data determined by these two methods was then used to determine an overall composite porosity for the respective disks. To do this the average and 2σ standard deviation from the individual cylinders from a particular disk was calculated. The average was determined to be the overall density of the disk. The final 2σ measurement uncertainty for the disk was the larger of (a) the largest 2σ measurement uncertainty of the three individual 2σ errors and (b) the 2σ standard deviation associated with the average value. These results are shown in Fig. 12, along with the bulk disk porosity determined from simple mass and volume measurements of the whole disks (Sec. 4.1). Table 14 shows the final measured porosities for all of the methods used in this paper. Figure 13 compares the individual cylinder porosity results for those cases where all three methods (mass/volume, Archimedes, XRCT) were applied.

In Figs. 11 and 12, especially for the higher porosity samples, the Archimedes value was always less than the bulk mass volume porosity. This makes sense if it is assumed that at the higher porosities, some of the pores were open to the surface, so that some water could have infiltrated the pore space, causing a smaller volume to be measured. This would give a higher density and therefore smaller porosity. Certainly, sample 1–5D showed that its pores were connected, and a quick visual inspection showed that there were open pores on the surface. Note that the bulk mass/volume ratios were measured before the Archimedes measurements were made, so that there was no water in the pore space.

### 4.7 Ultrasonic Wavespeed Results

**Fig. 14:**
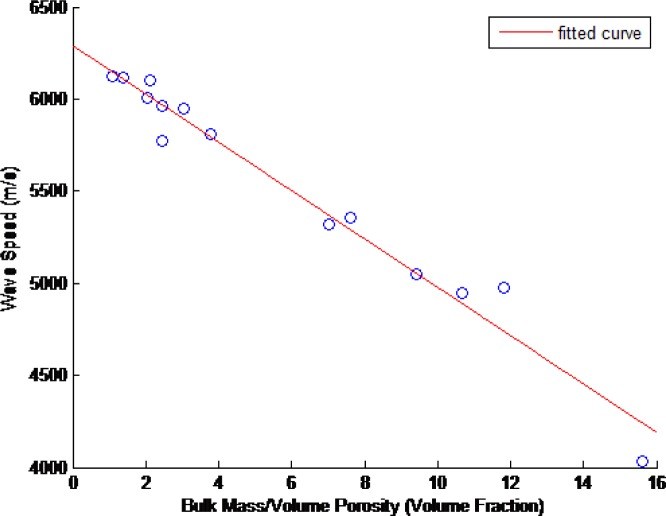
Measured ultrasonic wavespeeds as a function of measured porosity in the CoCr disks as determined by bulk mass and volume measurements.

**Fig. 15:**
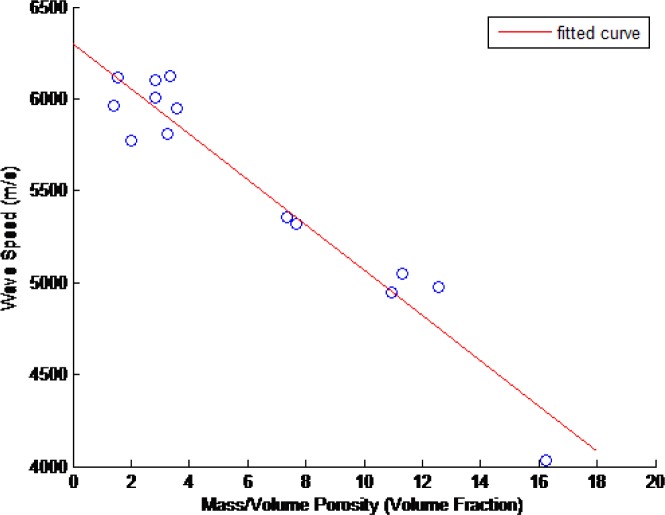
Measured ultrasonic wavespeeds as a function of measured porosity in the CoCr disks as determined from the mass and volume of the disks’ cylindrical samples (composite porosity.)

**Fig. 16:**
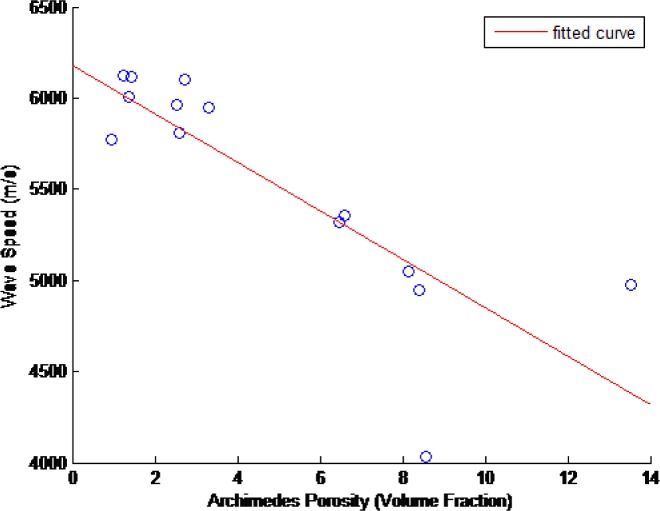
Measured ultrasonic wavespeeds as a function of measured porosity in the CoCr disks as determined from the disks’ cylindrical samples (composite porosity) using the Archimedes technique.

**Fig. 17:**
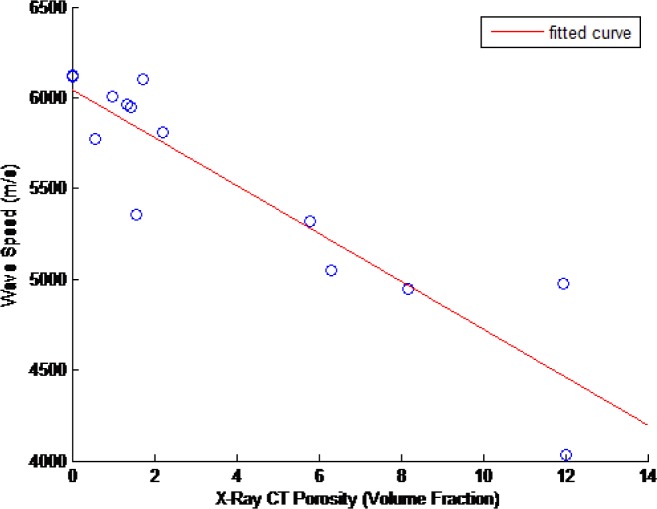
Measured ultrasonic wavespeeds as a function of measured porosity in the CoCr disks as determined by XRCT measurements on one cylinder taken from each disk.

The ultrasonic wavespeed of each of the CoCr disks was measured as described in Sec. 2. These results and their associated 2σ measurement uncertainties are shown in Table 15. Figures 14–17 show these measured ultrasonic wavespeeds (*v*) as a function of the measured disk porosity (*ϕ*) for each of the methods used here, and a fitted linear curve of the data [11–13,17] using the form

$$v=v_{o}+\beta \varphi$$ (8)

where *v_o_* is the ultrasonic wavespeed for a fully-dense specimen. Although the XRCT images show that the pores are neither spherical nor homogeneous, the elastic constants analysis in Sec. 4.5, and the fact that the overall porosity is low, justifies the use of a linear velocity-porosity relationship. Table 16 summarizes the fit correlation, slope (*β*), and predicted fully-dense wavespeed for each of the Fig. 14 – 17.

## 5. Sensor Design for *In-Situ* Porosity Measurements

The ultimate goal of this work is to incorporate an *in-situ* ultrasonic porosity sensor to detect changes in the porosity of a part while it is being fabricated in an additive manufacturing system. These changes may be an indication of unwanted process variability, and could potentially be used for real-time process modifications and control. Ultrasonic techniques and sensors such as the ones described in Sec. 2 and Sec. 4.7 are ideal for this application since they provide a rapid and non-destructive measurement while only requiring one-sided access to the specimen being measured.

### 5.1 Design Constraints

Three constraints inherent to the machine influenced our design for this *in-situ* sensor. These are the build process integrity, the build chamber environmental integrity, and temperature of the build plate.

While in operation, the machine consists of a number of moving parts. The recoater arm spreads each subsequent layer of metal powder by moving horizontally across the majority of the build chamber. The build plate, which supports the part being fabricated, moves in vertical steps to allow layer by layer powder deposition and fusion. Lastly, the powder bed (containing the metal powder) moves in vertical steps allowing the recoater arm to evenly remove its powder. Any design implemented on the inside of the machine cannot consist of wires or loose components that interfere with the motion of the machine’s critical parts. In addition, nothing inside the machine can obstruct the path of the laser while it is fusing metal powder.

The build chamber of the machine needs to maintain a certain environment while a build is being performed. The environment varies depending on the material being used for the build. CoCr is typically built in a Nitrogen environment that has an oxygen content of less than one percent. Any device inside the machine cannot affect the chemical composition of the atmosphere inside the build chamber. If the design consists of wiring that needs to come out of the machine, then the wires need to be small enough to come out of the door with the door shut or through a sealed ceiling port.

To prevent clumping of powder, the build plate is heated to 80 °C before a build commences, and this elevated temperature is maintained throughout a build. The ultrasonic sensor design should not have any impact on the overall temperature of the build chamber. In addition, the sensor and associated wiring should be protected against this elevated temperature.

### 5.2 Design

**Fig. 18:**
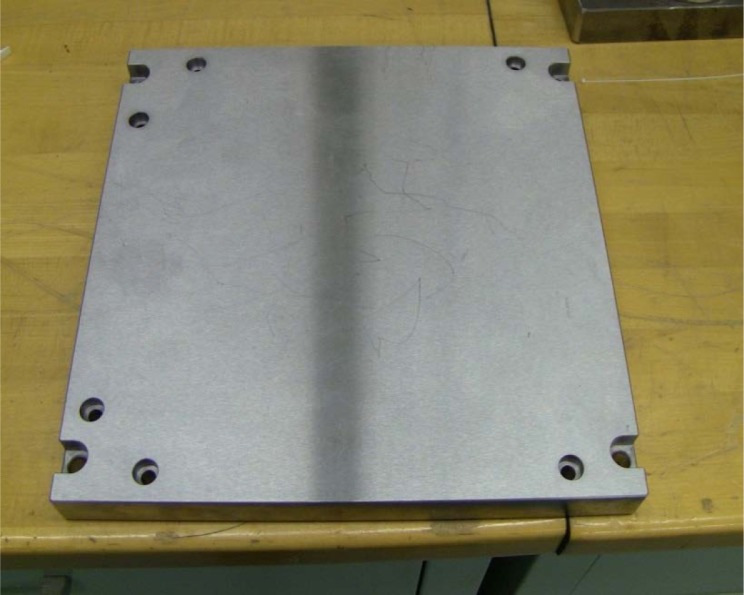
Typical 1045 Steel plate with dimensions 250 mm × 250 mm × 22 mm used for CoCr part production in an M270 DMLS system.

Figure 18 shows a typical 1045 steel build plate used for CoCr part production in the M270 DMLS system. This plate has approximate dimensions of 250 mm × 250 mm × 22 mm.

**Fig. 19:**
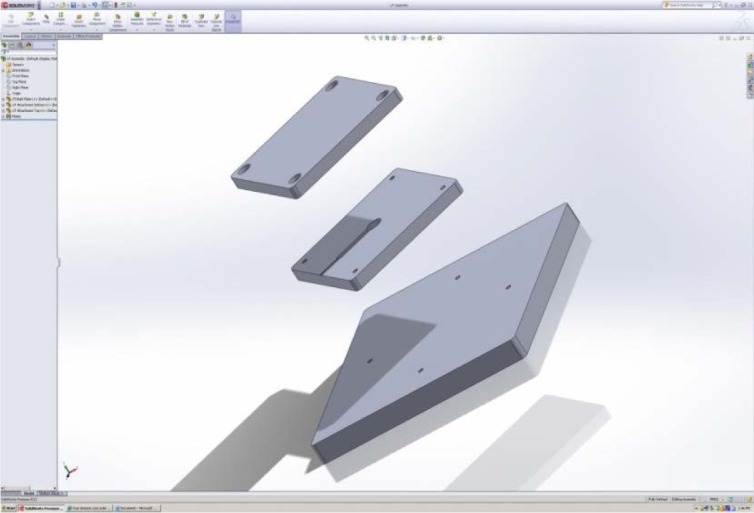
Two smaller build plates for housing ultrasonic porosity sensor.

**Fig.20:**
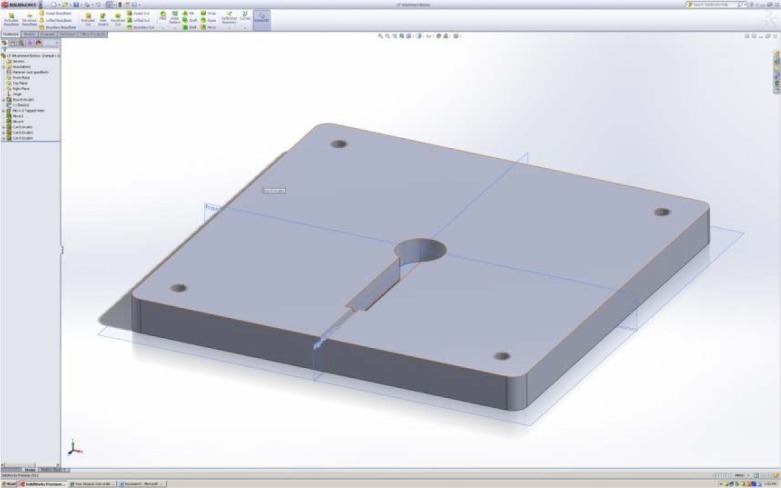
Lower build plate that houses ultrasonic transducer.

In our proposed design, two smaller build plates will be screwed onto the top of the primary build plate, as shown in Fig. 19. The lower of these two plates will house the ultrasonic porosity sensor (Fig. 20).

The designed attachment consists of two distinct pieces that connect to the build plate and each other via screws. The two pieces will encase the ultrasonic transducer and guide the wire out of the side through the small hole seen in Fig. 20. The overall dimensions of this attachment are 146 mm × 146 mm × 25 mm thickness. It is oriented in a diamond-like configuration which is preferred since it minimizes impacts with the recoater arm [36]. The material used for this design is the same as the material of the build plate (1045 Steel). This design places the ultrasonic sensor directly in the center of the build plate. However, if desired the lower build plate hole-position can be modified such that the ultrasonic measurements can be done at other locations.

The internal channel that forms when the two pieces are placed together is where the ultrasonic transducer will be placed. The hole has a diameter equal to that of the ultrasonic transducer (17.5 mm), and its depth is 8.5 mm into each side the attachment. This adds up to a total height of 17 mm, which is greater than the height of the ultrasonic transducer (about 16 mm). The reason for making the height of the channel greater than the height of the ultrasonic transducer is to assist with aligning and thermally insulating the ultrasonic transducer. A piece of putty or rubber that is larger than the 1 mm of free space should be placed into the bottom of the channel prior to the ultrasonic transducer. While the screws are being tightened this rubber or putty will compact and the ultrasonic transducer will be forced upward in contact with the top of the attachment, completely mating it with the top surface and wringing it with the required ultrasonic couplant. The rubber or putty will also provide thermal insulation from the heat source underneath the ultrasonic transducer. This insulation plus the offset distance from the top of the build plate to the bottom of the internal channel of the attachment should provide more than enough thermal protection for the ultrasonic transducer. The channel that extends from the hole is 4 mm thick, 40 mm long, and the same height as the hole. This part of the cutout is to hold the thicker part of the pulser/receiver wire connection to the ultrasonic transducer. Extra room is provided because upon tightening, the ultrasonic transducer will be moving into place and the wire cannot obstruct this motion. The small hole that further extends the channel to the outside of the attachment is there to guide the thin part of the wire out of the attachment so that data can be acquired. This part of the channel is designed to be a tight fit because the location of the wire leading out of the machine is critical to build process integrity.

The wire will come out of the attachment and run to the sidewall next to the recoater arm’s start position. Along this path, it will be attached to the build plate with tape or some other adhesive to prevent it from moving while it is buried in layers of powder. A small groove will be cut into the sidewall that allows the wire to pass under the recoater arm and into the collection chamber. There will be enough slack left so that the wire can move as far down as the build plate does during the build. The rest of the wire will be attached to the collection chamber again with tape or some adhesive and routed along the sides and out of the machine door. Data will be sent through this wire to an oscilloscope and digital aquisition will be done using a LabVIEW program that acquires the data (Sec. 5.4).

This design as a whole does not interfere with the build process integrity because it will not affect the motion of the machine’s critical parts. The wire, when routed correctly, will be able to move without interfering with the recoater arm or the build itself, all while extracting data through the machine door. In addition, this design will not affect the environmental integrity of the build chamber because none of the parts will generate or absorb a substantial amount of heat and the machine door will remain closed at all times.

### 5.4 Measurement Strategy

Most parts build by the DMLS process are complex, and would not be easily interrogated with ultrasonics while being built. As such, we plan to build a sacrificial part over the ultrasonic transducer, while the actual part being built is made on a different location of the build plate. This sacrificial part will be the same height as the actual part, will be cylindrical in size, and have a diameter of at least 17.5 mm (the same diameter as the ultrasonic transducer.) In this scheme, it is assumed that the part under test and the sacrificial part will have the same porosity.

For each layer, the time record (waveform) that shows the front- and back-wall echoes will be recorded, using customized LABView software and a digital oscilloscope. This recorded waveform will be an average of at least 512 acquisitions. The software will also calculate the time-of-flight between the front and back-wall echoes and calculate the velocity. Because the acquisition time of this measurement is so fast (Sec. 2), it will be possible to make the measurements during the time is takes for the M270 system to lay down the next layer of powder. The repeatable forming and known thickness of each layer also simplifies this measurement. The actual sensor integration into and measurements during a DMLS process are a planned next step.

## 6. Discussion and Conclusions

The porosity level in these parts was controlled by varying the build parameters in the DMLS AM process for each of the disks. Although the powder had the same properties for each build, it is possible that variable powder properties such as size distribution, morphology, and chemical composition could also have an effect on porosity. This will be an area of future work.

The different methods for measuring porosity presented here have different amounts of sophistication, and require different amounts of effort to do properly. The different methods give respective results that are slightly different, but they generally agree, especially when the measurement errors are taken into consideration. The results for the individual cylinders, when measured by the Archimedes and mass methods, generally agree, but there are some instances of discrepancy (Fig. 11). This discrepancy could be due to the buoyancy effects of water infiltration into surface breaking pores during the Archimedes measurements. Comparisons of the measured composite disk porosity, which was determined using Archimedes and the masses of the individual cylinders, as well and the masses and volumes of the disks, was also generally in consonance (Fig 12). However, the large error bars on the disks’ composite porosities determined from the individual cylinder measurements is an indication of the local differences in disk porosity that were inherent in the cylinders cut out of those disks. When all three porosity measurement methods (mass/volume, Archimedes, XRCT) are applied to the same individual cylinders, the agreement is quite good (Fig. 13).

The XRCT technique is particularly helpful in determining the porosity morphology and distribution. They reveal that the pores in these samples are often not spherical in shape. The XRCT also showed the presence of cracks, which sometimes cross the laser scan lines from the DMLS process. These cracks were routinely present for the samples that had a large amount of porosity. These samples with a large amount of porosity also tended to have pores that connected across many measured layers. Note however that the XRCT measured layers, which consisted of 2.4 um diameter voxels for the low resolution measurements, are only 10 % of the thickness of each melted layer (approximately 20 µm) deposited for each DMLS layer. It should be noted that in two cases (cylinders 1–1C and 3–1A) the XRCT measured zero porosity while the other methods measured a non-zero porosity of roughly 1 %–2 %. In these cases the measurement uncertainties did not include zero. This may be an indication of a lower limit in the size of porosity that can be detected with the XRCT technique.

It is difficult to conclude definitely which of these methods provides the true (e.g., “ground truth”) porosity value. Destructive methods would need to be done to ascertain this. Following all of the measurements reported in this paper, the disks were cut into two pieces, using a water-fed abrasive saw with a water-soluble machining coolant. The disks’ outer and interior cross-sectional surfaces were then examined using digital imaging techniques to confirm the presence of pores and to develop quantitative digital imaging measurements of porosity. These results will be presented at a later date.

The ultrasonic velocity and amount of porosity generally followed a linear correlation, despite the pores being neither spherical in shape nor homogeneously distributed (see Sec. 2). However, as shown in Sec. 4.5, at low amounts of porosity the linear relationship still holds true, and as the porosity increases, the shape of the pores become less spherical, and this combination of increased porosity with increasingly less spherical pores results in the linear relationship to remain true to a greater degree than reported previously.

Finally, the ultrasonic contact pulse-echo velocity measurement method appears sensitive enough to detect small changes (perhaps as small as 5 % absolute) in porosity. This should be sensitive enough to detect process deviations, *in-situ*, during a DMLS process.

## 7. Summary

In this paper we presented the development of well-characterized CoCr reference samples, built by varying the build parameters on a commercial DMLS AM system, and the application of several different measurement techniques for determining the porosity of these samples. These methods included Archimedes, as well as mass/volume, and XRCT. The porosity results generally agreed, and showed local variability of the porosity in each of the samples.

Ultrasonic pulse-echo velocity measurements were also applied to the samples, and a linear relationship between the ultrasonic velocity and the degree of porosity was shown. The sensitivity of the ultrasonic measurement is sensitive enough to detect small absolute changes (~ 0.5 %) in porosity. Elastic simulations to assess mechanical anisotropy were also performed, and were based on the XRCT results. These simulations showed that the linear dependence of the ultrasonic wavespeed on porosity measured here was reasonable, even for those cases where the porosity was large or non-spherical.

Finally a proposed sensor design and measurement strategy, for future experiments planned on a metal powder bed fusion system were presented.

## Figures and Tables

**Table 1:** Build parameters and preliminary measured porosity values for Build 1.

Sample	Hatch Speed (mm/s)	Hatch Spacing (mm)	Porosity by weight (%)	Porosity by micrograph (%)
1	800	0.1	0	0.07
2	1600	0.1	1.37	0.5
3	3200	0.1	18.12	12.22
4	800	0.2	2.07	0.29
5	800	0.4	10.19	14.38
6	3200	0.4	72.00	72.4

**Table 2:** Nominal physical and elemental abundance of parts made using EOS MP1 Cobalt Chrome Powder on a DMLS system using the standard EOS Cobalt Chrome parameter set [19].

Material Composition	Co (60 – 65 % by weight)
	Cr (26 – 30 % by weight)
	Mo (5 – 7 % by weight)
	Si (≤ 1.0 % by weight)
	Mn (≤ 1.0 % by weight)
	Fe (≤ 0.75 % by weight)
	C (≤ 0.16 % by weight)
	Ni (≤ .10 % by weight)

Relative Density	Approximately 100 %

Density	Approximately 8.3 g/cm^3^

**Table 3:** Measured bulk densities and porosities of CoCr disks.

Sample	Bulk density (g/cc)	2σ error (g/cc)	Bulk porosity (%)	2σ error (%, absolute)
1–1	8.21	0.10	1.10 %	0.01 %
1–2	8.10	0.09	2.44 %	0.03 %
1–4	8.12	0.10	2.13 %	0.03 %
1–5	7.32	0.09	11.82 %	0.14 %
2–1	8.10	0.11	2.45 %	0.03 %
2–2	7.41	0.10	10.68 %	0.14 %
2–3	7.67	0.14	7.63 %	0.13 %
2–4	7.52	0.10	9.41 %	0.13 %
2–5	7.00	0.09	15.60 %	0.21 %
3–1	8.19	0.09	1.37 %	0.02 %
3–2	8.13	0.09	2.06 %	0.02 %
3–3	8.05	0.09	3.03 %	0.03 %
3–4	7.99	0.09	3.78 %	0.04 %
3–5	7.72	0.08	7.04 %	0.08 %

**Table 4:** Measured densities and porosities of cylinders cut out of CoCr disks using the cylinders’ mass and volumes.

Sample	Density (g/cc)	2σ error (g/cc)	Porosity (%)	2σ error (%, absolute)
1–1A	8.15	0.42	1.79 %	0.09 %
1–1B	7.80	0.40	6.03 %	0.31 %
1–1C	8.11	0.42	2.27 %	0.12 %
1–2A	8.17	0.45	1.59 %	0.09 %
1–2B	8.17	0.45	1.59 %	0.09 %
1–2C	8.21	0.45	1.10 %	0.06 %
1–4A	7.78	0.40	6.31 %	0.32 %
1–4B	8.21	0.43	1.10 %	0.06 %
1–4C	8.21	0.43	1.10 %	0.06 %
1–5A	7.30	0.41	12.00 %	0.68 %
1–5B	7.30	0.41	12.00 %	0.68 %
1–5C	7.16	0.40	13.73 %	0.77 %
2–1A	8.20	0.41	1.25 %	0.06 %
2–1B	8.16	0.41	1.73 %	0.09 %
2–1C	8.05	0.40	3.05 %	0.15 %
2–2A	7.38	0.37	11.11 %	0.56 %
2–2B	7.40	0.37	10.90 %	0.54 %
2–2C	7.40	0.37	10.89 %	0.54 %
2–3A	7.77	0.39	6.36 %	0.32 %
2–3B	7.65	0.38	7.85 %	0.39 %
2–3C	7.65	0.38	7.86 %	0.40 %
2–4A	7.57	0.38	8.77 %	0.44 %
2–4B	7.06	0.35	14.97 %	0.74 %
2–4C	7.45	0.37	10.26 %	0.51 %
2–5A	7.03	0.35	15.25 %	0.77 %
2–5B	6.90	0.34	16.86 %	0.84 %
2–5C	6.92	0.35	16.59 %	0.83 %
3–1A	8.17	0.41	1.62 %	0.08 %
3–1B	8.26	0.41	0.52 %	0.03 %
3–1C	8.09	0.40	2.48 %	0.12 %
3–2A	8.00	0.40	3.60 %	0.18 %
3–2B	8.00	0.40	3.64 %	0.18 %
3–2C	8.19	0.41	1.31 %	0.07 %
3–3A	7.93	0.39	4.43 %	0.22 %
3–3B	8.12	0.41	2.13 %	0.11 %
3–3C	7.95	0.39	4.22 %	0.21 %
3–4A	0.00	0.00	0.00	0.00
3–4B	8.04	0.40	3.14 %	0.16 %
3–4C	8.02	0.40	3.32 %	0.17 %
3–5A	7.71	0.38	7.09 %	0.35 %
3–5B	7.78	0.39	6.22 %	0.31 %
3–5C	7.50	0.37	9.65 %	0.48 %

**Table 5:** Measured densities and porosities of cylinders cut out of CoCr disks using the Archimedes technique.^3^

Sample	Archimedes density (g/cc)	2σ error (g/cc)	Porosity (%)	2σ error (%, absolute)
1–1A	8.198	0.175	1.232 %	0.026 %
1–1B	8.198	0.175	1.232 %	0.026 %
1–1C	8.198	0.175	1.232 %	0.026 %
1–2A	8.091	0.250	2.521 %	0.078 %
1–2B	8.091	0.250	2.521 %	0.078 %
1–2C	8.091	0.250	2.521 %	0.078 %
1–4A	8.074	0.156	2.724 %	0.053 %
1–4B	8.074	0.156	2.724 %	0.053 %
1–4C	8.074	0.156	2.724 %	0.053 %
1–5A	7.178	0.388	13.521 %	0.730 %
1–5B	7.178	0.388	13.521 %	0.730 %
1–5C	7.178	0.388	13.521 %	0.730 %
2–1A	8.192	0.006	1.302 %	0.001 %
2–1B	8.261	0.006	0.468 %	0.000 %
2–1C	8.210	0.006	1.089 %	0.001 %
2–2A	7.573	0.006	8.759 %	0.007 %
2–2B	7.647	0.006	7.872 %	0.006 %
2–2C	7.588	0.006	8.577 %	0.007 %
2–3A	7.786	0.006	6.193 %	0.005 %
2–3B	7.820	0.006	5.784 %	0.004 %
2–3C	7.653	0.006	7.796 %	0.006 %
2–4A	7.707	0.006	7.140 %	0.006 %
2–4B	7.563	0.006	8.883 %	0.007 %
2–4C	7.603	0.006	8.395 %	0.006 %
2–5A	7.464	0.006	10.073 %	0.009 %
2–5B	7.683	0.007	7.429 %	0.007 %
2–5C	7.618	0.007	8.217 %	0.007 %
3–1A	8.175	0.006	1.510 %	0.001 %
3–1B	8.139	0.005	1.939 %	0.001 %
3–1C	8.225	0.006	0.900 %	0.001 %
3–2A	8.192	0.006	1.298 %	0.001 %
3–2B	8.214	0.006	1.033 %	0.001 %
3–2C	8.156	0.006	1.738 %	0.001 %
3–3A	8.172	0.006	1.543 %	0.001 %
3–3B	7.815	0.005	5.841 %	0.004 %
3–3C	8.091	0.006	2.524 %	0.002 %
3–4A	8.094	0.006	2.483 %	0.002 %
3–4B	8.115	0.006	2.228 %	0.002 %
3–4C	8.042	0.006	3.113 %	0.002 %
3–5A	7.805	0.006	5.958 %	0.004 %
3–5B	7.746	0.006	6.677 %	0.005 %
3–5C	7.738	0.006	6.785 %	0.005 %

**Table 6:** Porosity results for all 14 test samples using X-ray CT measurements.

Sample	Number of Slices	Pixel size (μm)	Average porosity (%)	Porosity Standard Deviation (Absolute, %)
1–1C	900	2.447	0.000	0.000

1–2C	906	2.447	1.348	0.262
1–2D	851	2.540	1.305	0.268
1–4C	909	2.430	1.720	0.143
1–5C	776	2.524	11.960	1.190
1–5D	801	2.440	11.230	1.390
2–1C	908	2.447	0.571	0.348
2–2A	903	2.447	8.191	2.340
2–3A	909	2.447	1.569	0.290
2–4A	901	2.447	6.313	1.702
2–5A	916	2.447	12.020	2.660

3–1A	900	2.447	0.000	0.000
3–2A	906	2.447	0.971	0.195
3–3A	905	2.447	1.447	0.275
3–4A	921	2.626	2.199	0.456
3–5A	906	2.447	5.778	0.802

**Table 7:** Information about the four sets of X-ray CT scans that were carried out on two of samples from the three sample sets.

Sample	Voxel size	Image size	Number of images	Average porosity and standard deviation (%) in z direction for entire image set	Threshold for pore-solid (gray scale)
1–5D	0.87 µm	984 × 1009	851	10.650 ± 2.550	32 000
1–5D	2.44 µm	978 × 979	801	11.230 ± 1.390	32 000
1–2D	0.82 µm	976 × 1012	851	0.895 ± 0.434	32 000
1–2D	2.54 µm	976 × 978	851	1.305 ± 0.268	40 000

**Table 8:** Average and standard deviation porosity for 620 × 620 × full depth microstructure piece.

Sample	Voxel size	Average Porosity	Standard Deviation in x-Direction (%)	Standard Deviation in y-Direction (%) *σ_y_*	Standard Deviation in Standard Deviation
1–5	0.87 µm	10.13	2.21	4.38	3.97
1–5	2.44 µm	11.37	1.57	1.25	1.75
1–2	0.82 µm	1.11	0.35	0.46	0.48
1–2	2.54 µm	1.08	0.19	0.19	0.32

**Table 9:** The average and standard deviation of the x, y, and z direction lengths of the pores in the sample 1-2D scans, at both resolutions.

Sample	Resolution (µm/voxel)	X-Direction Porosity Average and Standard Deviation (µm)	Y-Direction Porosity Average and Standard Deviation (µm)	Z-Direction Porosity Average and Standard Deviation (µm)
1–2	0.82	89.7 ± 73.9	92.8 ± 72.0	73.2 ± 40.6
1–2	2.54	70.4 ± 49.0	70.6 ± 50.9	62.6 ± 33.1

**Table 10:** The computed elastic moduli tensor (C_ij_) for a 400^3^ piece of the sample 1–5D microstructure, high resolution, in units of GPa. The porosity of this microstructure piece was 9.8 %, close to the full value obtained on the complete set of scans. After spherical averaging, K = 85.3 GPa, G = 55.7 GPa, E = 137.2 GPa, *v* = 0.232.

C_ij_	1	2	3	4	5	6
**1**	166.4	49.13	45.98			
**2**	49.13	169.5	45.11			
**3**	45.98	45.11	150.9			
**4**				53.98		
**5**					51.76	
**6**						57.11

**Table 11:** The computed elastic moduli tensor (C_ij_) for a 400^3^ piece of the sample 1–5D microstructure, low resolution, in units of GPa. The porosity of this microstructure piece was 10.5 %, close to the full value obtained on the complete set of scans. After spherical averaging, K = 90.5 GPa, G = 58.4 GPa, E = 144.2 GPa, *v* = 0.235.

C_ij_	1	2	3	4	5	6
**1**	170.4	52.09	51.22			
**2**	52.09	167.2	50.96			
**3**	51.22	50.96	168.4			
**4**				58.28		
**5**					57.99	
**6**						58.49

**Table 12:** The computed elastic moduli tensor (C_ij_) for a 400^3^ piece of the sample 1–2D microstructure, high resolution, in units of GPa. The porosity of this microstructure piece was 0.99 %, close to the full value obtained on the complete set of scans. After spherical averaging, K = 129.0 GPa, G = 79.0 GPa, E = 194.7 GPa, *v* = 0.248.

C_ij_	1	2	3	4	5	6
**1**	233.04	77.06	76.89			
**2**	77.06	233.19	76.92			
**3**	76.89	76.92	232.63			
**4**				77.92		
**5**					77.96	
**6**						78.02

**Table 13:** The computed elastic moduli tensor (C_ij_) for a 400^3^ piece of the sample 1–2D microstructure, low resolution, in units of GPa. The porosity of this microstructure piece was 1.19 %, close to the full value obtained on the complete set of scans. After spherical averaging, K = 128.7 GPa, G = 77.8 GPa, E = 194.1 GPa, *v* = 0.248.

C_ij_	1	2	3	4	5	6
**1**	232.76	76.99	76.89			
**2**	76.99	232.89	76.92			
**3**	76.73	76.74	231.46			
**4**				77.65		
**5**					77.67	
**6**						77.88

**Table 14:** Final measured porosities for all of the methods used in this paper.

	Mass/Vol Porosity (%)	2σ error (%, absolute)	Archimedes Porosity (%)	2σ error (%, absolute)	XRCT Porosity (%)	1σ error (%,absolute)
						
**1**–**1**	**1.10 %**	**0.01 %**				
1–1A	1.79 %	0.09 %	1.23 %	0.03 %		
1–1B	6.03 %	0.31 %	1.23 %	0.03 %		
1–1C	2.27 %	0.12 %	1.23 %	0.03 %	0.00	0.00
**composite**	**3.36 %**	**4.6445 %**	**1.23 %**	**0.03 %**		
**1**–**2**	**2.44 %**	**0.03 %**				
1–2A	1.59 %	0.09 %	2.52 %	0.08 %		
1–2B	1.59 %	0.09 %	2.52 %	0.08 %		
1–2C	1.10 %	0.06 %	2.52 %	0.08 %	1.35	0.26
**composite**	**1.43 %**	**0.56 %**	**2.52 %**	**0.08 %**		
**1**–**4**	**2.13 %**	**0.03 %**				
1–4A	6.31 %	0.32 %	2.72 %	0.05 %		
1–4B	1.10 %	0.06 %	2.72 %	0.05 %		
1–4C	1.10 %	0.06 %	2.72 %	0.05 %	1.72	0.14
**composite**	**2.84 %**	**6.02 %**	**2.72 %**	**0.05 %**		
**1**–**5**	**11.82 %**	**0.14 %**				
1–5A	12.00 %	0.68 %	13.52 %	0.73 %		
1–5B	12.00 %	0.68 %	13.52 %	0.73 %		
1–5C	13.73 %	0.77 %	13.52 %	0.73 %	11.96	0.69
**composite**	**12.58 %**	**2.00 %**	**13.52 %**	**0.73 %**		
**2**–**1**	**2.45%**	**0.03%**				
2–1A	1.25 %	0.06 %	1.30 %	0.0010 %		
2–1B	1.73 %	0.09 %	0.47 %	0.0003 %		
2–1C	3.05 %	0.15 %	1.09 %	0.0008 %	0.571	0.349
**composite**	**2.01 %**	**1.86 %**	**0.95 %**	**0.87 %**		
**2**–**2**	**10.68%**	**0.14%**				
2–2A	11.11 %	0.55 %	8.76 %	0.007 %	8.19	2.34
2–2B	10.90 %	0.54 %	7.87 %	0.006 %		
2–2C	10.89 %	0.54 %	8.58 %	0.007 %		
**composite**	**10.9670 %**	**0.5560 %**	**8.4026 %**	**0.9368 %**		
**2**–**3**	**7.63 %**	**0.13 %**				
2–3A	6.36 %	0.32 %	6.19 %	0.005 %	1.57	0.29
2–3B	7.85 %	0.39 %	5.78 %	0.004 %		
2–3C	7.86 %	0.40 %	7.80 %	0.006 %		
**composite**	**7.36 %**	**1.73 %**	**6.59 %**	**2.13 %**		
**2**–**4**	**9.14 %**	**0.13 %**				
2–4A	8.77 %	0.44 %	7.14 %	0.006 %	6.31	1.70
2–4B	14.97 %	0.74 %	8.88 %	0.007 %		
2–4C	10.26 %	0.51 %	8.39 %	0.006 %		
**composite**	**11.33 %**	**6.47 %**	**8.13 %**	**1.80 %**		
**2**–**5**	**15.60 %**	**0.21 %**				
2–5A	15.25 %	0.77 %	10.07 %	0.009 %	12.02	2.66
2–5B	16.86 %	0.84 %	7.43 %	0.007 %		
2–5C	16.59 %	0.83 %	8.22 %	0.007 %		
**composite**	**16.23 %**	**1.72 %**	**8.57 %**	**2.71 %**		
**3**–**1**	**1.37 %**	**0.02 %**				
3–1A	1.62 %	0.08 %	1.51 %	0.001 %	0.00	0.00
3–1B	0.52 %	0.03 %	1.94 %	0.001 %		
3–1C	2.48 %	0.12 %	0.90 %	0.001 %		
**composite**	**1.54 %**	**1.97 %**	**1.45 %**	**1.04 %**		
**3**–**2**	**2.06 %**	**0.02 %**				
3–2A	3.60 %	0.18 %	1.30 %	0.001 %	0.97	0.20
3–2B	3.64 %	0.18 %	1.03 %	0.001 %		
3–2C	1.31 %	0.07 %	1.74 %	0.001 %		
**composite**	**2.85 %**	**2.66 %**	**1.36 %**	**0.71 %**		
**3**–**3**	**3.03 %**	**0.03 %**				
3–3A	4.43 %	0.22 %	1.54 %	0.001 %	1.45	0.28
3–3B	2.13 %	0.11 %	5.84 %	0.004 %		
3–3C	4.22 %	0.21 %	2.52 %	0.004 %		
**composite**	**3.59 %**	**2.540 %**	**3.30 %**	**4.50 %**		
**3**–**4**	**3.78 %**	**0.04 %**				
3–4A	0.00	0.00	2.48 %	0.002 %	2.199	0.456
3–4B	3.14 %	0.16 %	2.23 %	0.002 %		
3–4C	3.32 %	0.17 %	3.17 %	0.002 %		
**composite**	**3.23 %**	**0.27 %**	**2.61 %**	**0.91 %**		
**3**–**5**	**7.04 %**	**0.08 %**				
3–5A	7.09 %	0.35 %	5.96 %	0.004 %	5.78	0.80
3–5B	6.22 %	0.31 %	6.68 %	0.005 %		
3–5C	9.65 %	0.48 %	6.78 %	0.005 %		
**composite**	**7.65 %**	**3.57 %**	**6.47 %**	**0.90 %**		

**Table 15:** Measured ultrasonic wavespeeds and associated 2σ measurement uncertainties for each of the CoCr disks.

Sample	Wavespeed (m/s)	2σ error (m/s)
1–1	6123	79
1–2	5961	76
1–4	6099	79
1–5	4976	61
2–1	5773	80
2–2	4945	128
2–3	5357	70
2–4	5047	81
2–5	4035	91
3–1	6118	80
3–2	6008	78
3–3	5947	77
3–4	5809	75
3–5	5317	67

**Table 16:** Summary of the fit correlation and predicted fully-dense wavespeed for each of the Figs. 13–16.

	Bulk Mass/Volume Porosity	Composite Mass/Volume Porosity	Archimedes Porosity	X-ray CT Porosity
**Correlation R^2^**	0.9676	0.9295	0.6700	0.8265
***v****_o_* **(m/s)**	6292 (m/s)	6303 (m/s)	6181 (m/s)	6047 (m/s)
***β* (m/s/%)**	−131.2 (m/s/%)	−123.3 (m/s/%)	−132.9 (m/s/%)	−132.0 (m/s/%)

## Footnotes

1 Certain commercial equipment, instruments, or materials are identified in this paper to foster understanding. Such identification does not imply recommendation or endorsement by the National Institute of Standards and Technology, nor does it imply that the materials or equipment identified are necessarily the best available for the purpose.

2 The value of *d*(*x*) is defined such that a volumetric fraction x of the particles had a measured diameter of *d* or less.

3 Note that the measured values for set 1 repeat since each cylinder was measured once and the result is the average of those measurements. In set 2 and 3 each cylinder was measured three times.

## 8. Appendix A: Summary of the Methods Used To Determine the Measurement Errors Reported In This Paper

In consonance with NIST policy, all measurement uncertainties reported here are expanded uncertainties with a k = 2 coverage factor [21]. Since the uncertainties presented in this paper include both Type-A uncertainties (e.g., those uncertainties that are determined by statistical methods) and Type-B uncertainties (e.g., those uncertainties that are determined by other means), some additional detail is provided in this appendix.

The absolute error of the disk and cylinder volumes can be found in a straightforward way by taking the differential of the equation for a cylindrical volume, with the result shown in Eq. (A1).

$$\Delta V=V\left[\frac{2\Delta d}{d}+\frac{\Delta h}{h}\right]$$ (A1)

Here *V* is the calculated cylindrical volume, *d* is the measured cylinder diameter (average of three independent measurements), *h* is the measured cylinder height (average of three independent measurements), and Δ*d* and Δ*h* are the calculated standard deviations of the diameter and height measurements, respectively.

The density of each cylinder as measured by weighing also has a straightforward absolute error by taking the differential of the relationship between density, mass, and volume, as shown in Eq. (A2)

$$\Delta \rho =\rho \left[\frac{\Delta M}{M}+\frac{2\Delta d}{d}+\frac{\Delta h}{h}\right]$$ (A2)

where *M* is the measured cylinder mass (average of three independent measurements) and Δ*M* is the calculated standard deviation of the mass measurements.

For the Archimedes measurements, the measured cylinder density is given by

$$\rho =\left(\frac{M_{a}}{M_{a}-M_{w}}\right)\rho_{W}$$ (A3)

where *M_a_* is the measured mass of the cylinder (average of three independent measurements) measured in air, *M_w_* is the measured mass of the cylinder (average of three independent measurements) measured in water, and *ρ_W_* is the density of water, which we assume to be 1.0 g/cm^3^ (see Sec. 4.4). Taking the differential to determine the absolute error results in a rather complicated expression:

$$\Delta \rho =\rho \left[\frac{\Delta M_{a}{\left(M_{a}-M_{w}\right)}^{-1}-M_{a}{\left(M_{a}-M_{w}\right)}^{-2}(\Delta M_{a})}{M_{a}}+\frac{M_{a}{\left(M_{a}-M_{w}\right)}^{-2}(\Delta M_{w})}{M_{w}}\right]$$ (A4)

Fortunately in this case the instrument reading error Δ is the same for both measurements made in water and measurements made in air. Thus Δ*M_a_* = Δ*M_w_* = Δ, and Eq. (A4) can be simplified to a much more compact expression:

$$\Delta \rho =\rho \left[\frac{\Delta }{{\left(M_{a}-M_{w}\right)}^{2}}\right]\;\left[\frac{M_{a}}{M_{w}}-\frac{M_{w}}{M_{a}}\right]$$ (A5)

To arrive at the final disk density from the individual density measurements of its respective cylinders, the following procedure was used:

1. The average and 2*σ* standard deviation for the three cylinders were calculated.
2. The disk density is the average of these three cylinders.
3. The 2*σ* is the larger of either (a) the largest of the three individual cylinder 2*σ* errors; and (b) the 2*σ* standard deviation corresponding to the average value.
